# T cell-specific HIF-2α attenuates colitis by antagonizing notch-driven Th2 differentiations

**DOI:** 10.3389/fimmu.2026.1755068

**Published:** 2026-03-03

**Authors:** Ting Gao, Liangfeng Gao, Hui Zhang, Zaizhi Liu, Qing Zhu, Chunyan Wang, Song Zhang, Nan Feng

**Affiliations:** 1 Emergency Department, Ren Ji Hospital, School of Medicine, Shanghai Jiao Tong University, Shanghai, China; 2 Department of Cardiology, Zibo Central Hospital, Zibo, Shandong, China

**Keywords:** HIF-2α, lymphocytes, notch signaling pathway, Th2 differentiation, ulcerative colitis

## Abstract

**Objective:**

The function of hypoxia-inducible factor-2α (HIF-2α) in ulcerative colitis pathogenesis is subject to ongoing debate, with conflicting reports indicating either disease-promoting or beneficial roles. This investigation was designed to define the precise contribution of T lymphocyte-restricted HIF-2α to UC development.

**Methods:**

We immunohistochemically stained colonic biopsy tissues from human UC donors and healthy controls. A Lck-Cre-mediated Cre-loxP mediated HIF-2α conditional knockout mouse (HIF-2^ΔT/NKT) was generated and subjected to DSS-induced colitis induction. For mechanistic experiments, primary CD4^+^ T cells were transduced with lentiviral vectors harboring HIF-2-directed shRNA or cDNA. Protein-Protein interactions, signal transduction and cellular phenotype were assessed by Co-IP, qPCR array, multi-parametric flow cytometry, immunoblotting and histology.

**Results:**

Immunohistochemistry demonstrated increased HIF-2α expression in colonic tissues from UC patients, but its expression levels in lymphocyte subtypes were inversely correlated with the clinical disease severity. Mice with T/NKT cell specific HIF-2 deletion were more susceptible to DSS colitis, associated with abrogated polarization of T helper cells towards the Th2 lineage. This led to upregulation of *Il4* and *Gata3* transcription, as well as increased production of IL-4 cytokine. Molecularly, HIF-2α bound directly to the proteolytically cleaved form of Notch1 intracellular domain (NICD), leading to a decrease in NICD-induced transcriptional activity. Consistent with this mode of action, forced HIF-2α expression in CD4^+^ T cells abrogatedJagged1-induced Th2 commitment *in vitro*. In contrast, HIF-2α deficiency enhanced Notch pathway signaling, promoted Th2 polarization and exacerbated colonic inflammation *in vivo*.

**Conclusion:**

Our results establish a non-redundant protective function for T cell-intrinsic HIF-2α in ulcerative colitis. The protein directly engages Notch1-NICD to restrain Notch signal transduction, ultimately limiting pathological Th2 cell differentiation and ameliorating intestinal inflammation. These data reconcile prior contradictory findings by revealing a crucial cellular context-dependent mechanism.

## Introduction

Inflammatory bowel disease (IBD), consisting of ulcerative colitis (UC) and Crohn’s disease, is a group of chronic, immune-mediated inflammatory disorders of the digestive tract (1, 2). The exact cause of this chronic inflammation in the gut, which is characteristic to both UC and Crohn’s, is still not fully clear (3, 4). In addition to genetic predisposition, environmental factors are known to be contributors, it is still unknown for the mechanistic roles in the disease flares (5). Tissue hypoxia is a major pathophysiological hallmark of IBD, but it is manifested by multi-etiology and not yet clearly understood (6–8). Among implicated contributors is increased oxygen consumption by metabolically active epithelial cells during inflammation, reduced blood perfusion as a consequence of microvasculitis, and significant oxygen utilization by migrating neutrophils that jointly exacerbate hypoxic stress in the intestinal mucosa (9–11).

The hypoxia-inducible factors are key regulators of cellular adaptation under hypoxic conditions, which form heterodimeric transcription factors consisting of an oxygen-labile α-subunit and a constitutively expressed β-subunit (12, 13). These master regulators regulate the transcription of many downstream genes and allow for physiological adaptations to hypoxia (14, 15). HIF-1α and HIF-2α Both Exist in IBD Patients The downstream functions of such two factors in intestinal epithelia of IBD patients seem to be different (16–21). The role of HIF-2α has been especially intricate and controversial, as different studies have revealed contrary findings. For example, overexpression of HIF-2 in the intestinal epithelial compartment was found to enhance chemically induced colitis in mice, while in other models its gene expression increased seems to be protective against radiation-induced mucosal injury (18, 21, 22), thus the concept about whether it functions as pro-inflammatory or reparative factor remain uncertain.

Moreover, the activation of HIF-1α in inflamed mucosa is functionally linked to the upregulation of barrier protective genes and -defensins thereby augmenting innate immunity and antimicrobial defense in a hypoxia- and HIF-1α-dependent manner (23, 24). These are protective measures in colitis commonly thought. In contrast, HIF-2α is critically involved in regulating epithelial inflammatory responses; its chronic activation can potentiate pro-inflammatory signaling, exacerbate intestinal damage, and potentially increase cancer risk (25–27). The complex cooperation and context dependent regulation of these two HIF-α variants in inflamed mucosa merit further studies.

Here we explore the role of HIF-2α in ulcerative colitis, with a particular focus on its effects on T cell biology. Through immunohistochemical measurements of mucosal and submucosal sections from UC patients, an inverse relationship between HIF-2α expression in lymphocytes and the extent of damage to the intestine could be observed. Using T/NKT cell-specific HIF-2α conditional knockout (T/NKT-HIF-2α cKO) mice mediated by Lck-Cre, we assessed the impact of this loss on dextran sulfate sodium (DSS)-induced colitis. Our data demonstrate that T/NKT-HIF-2α cKO mice develop more severe intestinal pathology, suggesting a protective role for T cell-intrinsic HIF-2α during inflammation. In addition, lentiviral-based modulation of HIF-2α expression in T cells demonstrated that it acts as a negative regulator of Th2 differentiation. Altogether, we reveal here HIF-2α as an important regulator of Th2 responses and provide a foundation for its targeting in immunotherapy of UC.

## Methods

### Human tissue samples

Patients Biopsy specimens of the intestine were obtained from fifteen patients with active UC. Classification of disease severity into mild and severe categories was based on standardized clinical and endoscopic methods. Controls were obtained from subjects undergoing screening colonoscopy with no identifiable lesions. The experimental use with all human samples obtained formal approval from the Institutional Review Board of Ren Ji Hospital of Shanghai, China. Moreover, all participants gave their written informed consent before they were included in this study.

### Immunohistochemistry

Mucosal and submucosal samples were derived from the intestines of both ulcerative colitis patients and healthy control individuals. The same procedures were performed in experimental mice for collecting colonic tissue. All obtained samples were fixed in 4% paraformaldehyde and embedded in paraffin blocks. The blocks were cut into serial sections of standardized thickness (5 μm). For immunohistochemical analysis, human tissue sections were probed with a specific antibody targeting HIF-2α (Abcam ab243681), while murine sections were incubated with an anti-GATA3 antibody (Abcam ab199428). Following primary antibody application, all sections were treated with appropriate secondary antibodies, visualized using diaminobenzidine (DAB) substrate, and subsequently counterstained with hematoxylin. Protein expression levels of HIF-2α were quantitatively assessed through a standardized immunohistochemical scoring methodology. The scoring criteria (28) are as follows: 0 points: no expression; 1 point: weak expression (≤10% of cells positive); 2 points: moderate expression (10%-30% of cells positive); 3 points: moderately strong expression (30%-50% of cells positive); 4 points: strong expression (>50% of cells positive).

### Mice and genotyping

We generated T/NKT cell-specific HIF-2α conditional knockout (T/NKT-HIF-2α cKO) mice by crossing *Hif2a*^flox/flox^ mice with *Lck-Cre* transgenic mice. The generation steps of T/NKT-HIF-2α cKO mice (**Supplementary Figure 1**) is as follows:

Step 1: Parental Mice Preparation

Mouse A: *Hif2a* flox/flox (Homozygous for floxed allele)

Mouse B: *Lck-Cre* (Transgenic for T-cell-specific Cre recombinase)

Step 2: Initial Cross (to generate F1 carriers)

*Hif2a* flox/flox × *Lck-Cre*

↓

F1 Offspring: Genotype *Hif2a* flox/+; *Lck-Cre*+

Step 3: Breeding & Selection (Backcross to obtain homozygous floxed mice)

Cross: F1 (*Hif2a* flox/+; *Lck-Cre*+) × *Hif2a* flox/flox

↓

Offspring (Multiple Genotypes):

Target Experimental Group: *Hif2a* flox/flox; *Lck-Cre*+ (T/NKT cKO)

Littermate Control Group: *Hif2a* flox/flox; *Lck-Cre*- (Wild-type control, WT)

Step 4: Genotype Confirmation

Perform PCR on tail/ear DNA to verify

All mice were housed in specific pathogen-free environments, and all animal experiments followed the protocols approved by the Animal Care and Use Committee of Shanghai Tenth People’s Hospital.

### Induction of colitis

Experimental colitis was induced in 8-week-old male mice through administration of drinking water containing 2.5% (w/v) DSS (Sigma-Aldrich, MW: 36,000-50,000) for seven consecutive days. Throughout the induction period, body weight and Disease Activity Index (DAI) was monitored daily as a key clinical parameter. DAI score = (score of weight loss) + (score of stool consistency) + (score of hematochezia). weight loss (0-4, 0 to 15% loss), stool consistency (0 points for normal, 2 points for loose stool, and 4 points for diarrhea) and hematochezia (0 points for normal, 2 points for positive occult blood, and 4 points for overt hemorrhage). On day 7 post-induction, animals were euthanized for tissue collection, where colon length was measured as a primary indicator of disease severity. The harvested colonic tissues were subsequently processed for comprehensive histological examination and molecular characterization. For euthanasia of mice, each animal was first placed in a sealed chamber filled with room air. Carbon dioxide (CO_2_) was then introduced at a flow rate of 20% of the chamber volume per minute to raise the CO_2_ concentration to 40% within 2 min, thereby inducing anesthesia. The flow rate was subsequently increased rapidly (40% per minute) to elevate the CO_2_ concentration to 80% in the third minute, which was maintained for 3–5 min to ensure complete cardiac arrest. Immediately after complete loss of consciousness, cervical dislocation was performed. Multiple criteria for death confirmation were assessed, including absence of spontaneous respiration and heartbeat, fixed and dilated pupils, and lack of response to a painful stimulus.

### Cell isolation and lentiviral transduction

Primary human CD4^+^ T lymphocytes were isolated from healthy donor peripheral blood mononuclear cells by negative selection using a commercial CD4^+^ T Cell Isolation Kit (Thermo, 11352D) according to the manufacturer’s instruction. CD4^+^ T cells were cultured in RPMI 1640 complete medium supplemented with 10% fetal bovine serum (FBS) for 5 days. The culture plates were coated with anti-CD3 antibody (5 μg/mL), and anti-CD28 antibody (1 μg/mL) along with IL-2 (10 ng/mL) were added to the medium to provide T cell activation signals. At day 5, harvest the cells and resuspend them in fresh complete medium containing a low dose of IL-2 (1 ng/mL), and culture overnight (16 h) in a culture plate (no coating required). For HIF-2α modulation, the monolayer cells were transduced by lentiviral vector carrying either HIF-2α-targeting short hairpin RNA (shRNA) for gene knockdown or a full-length HIF-2α coding sequence for constitutive overexpression.

### Flow cytometry

For restimulation and inhibitor treatment: Cells were harvested and restimulated with phorbol 12-myristate 13-acetate (PMA, 50 ng/mL) and ionomycin (1 µg/mL) at 37 °C for 4 h. At the start of stimulation, a protein transport inhibitor (Brefeldin A, 5 µg/mL, 4h) was added to prevent newly synthesized IL-4 from being secreted extracellularly. After stimulation, cells were first stained for surface CD4 using a fluorochrome-conjugated antibody (Thermo, 11-0049-42). Subsequently, the cells were fixed (with 0.4% paraformaldehyde) and permeabilized (with Triton X-100). Following permeabilization, intracellular IL-4 was stained using a fluorochrome-conjugated anti-IL-4 antibody (Thermo, 12-7049-42). Finally, the fully stained cells were analyzed on a Cytoflex flow cytometer. The CD4^+^ lymphocyte population was gated, and the proportion of IL-4^+^ cells within this population was quantified using FlowJo software. The flow cytometry gating strategy diagram was shown in **Supplementary Figure 2**.

### Quantitative real-time PCR and PCR array

Total RNA was extracted from colonic mucosa tissue samples or *in vitro* cultured cells utilizing TRIzol reagent. Complementary DNA (cDNA) synthesis was then generated by a commercial Reverse Transcription Kit (Vazyme, R323-01). Gene expression was quantified using quantitative real-time PCR (qRT-PCR) with SYBR Green master mix (Vazyme, R321-02) on a QuantStudio platform. A pre-configured PCR array specific for the major genes of T helper cell differentiation (Th1, Th2, Th17) and TLR signaling pathways was also tested as recommended by the manufacturer.

### Western blotting

RIPA lysis buffer was used to extract protein lysates from colonic tissue or purified CD4^+^ T cells. Protein levels were determined with a BCA assay kit (Vazyme, E112-01). The western blots were then processed through dehydration, electrophoresis, transfer and blocking. And the membranes were incubated overnight with specific primary antibodies targeting anti-Notch1 (Abcam, ab52627), anti-Notch2 (Abcam, ab307700), Anti-NICD (Abcam, ab8387), Anti-RBPJK (Abcam, ab180588), Anti-Hes1 (Abcam, ab71559), Anti-HIF-2α (Abclonal, A7553) and Anti-GATA3 (Abcam, ab282110), followed by incubation with appropriate secondary antibodies. GAPDH (Abclonal, A19056) served as the loading control for normalization. Protein bands were ultimately visualized employing an iBright imaging system (Thermo).

### Enzyme linked immunosorbent assay

Cytokine concentrations for IL-4 (JONLNBIO, JL19287; COIBO, CB10185), IL-1β (JONLNBIO, JL18442), IL-17 (JONLNBIO, JL20250), and IFN-γ (JONLNBIO, JL10967) in murine serum samples and cell culture supernatants were quantified using commercially available ELISA kits, with all procedures conducted in strict adherence to the manufacturers’ instructions.

### GST (glutathione S-transferase) pull-down assay

To generate a GST fusion protein, the human HIF-2α coding sequence was cloned into a pGEX-4T-1 vector. Recombinant GST-HIF-2α and GST control proteins were expressed in Escherichia coli and subsequently affinity-purified using glutathione-Sepharose beads. Purified beads bound to either GST or the fusion protein were incubated with the NICD produced by GenePharma. After extensive washing to remove non-specifically bound material, proteins retained on the beads were eluted and detected by immunoblotting with a Notch1-specific antibody.

### Histological analysis

For histological examination, colonic specimens were fixed in 4% paraformaldehyde, embedded in paraffin, and sectioned. Tissue sections underwent hematoxylin and eosin (H&E) staining for general pathological assessment, with subsequent scoring based on the severity of inflammatory infiltration, architectural damage, and crypt loss. Additionally, Alcian Blue/Periodic Acid-Schiff (AB-PAS) staining was performed to identify goblet cells and enable their quantitative evaluation.

### Statistical analysis

Results are presented as mean ± standard deviation (SD). Group comparisons were performed using either one-way ANOVA followed by Tukey’s *post-hoc* analysis or unpaired Student’s t-test, implemented in GraphPad Prism software (version 9.0). The following thresholds defined statistical significance: *p < 0.05, **p < 0.01, ***p < 0.001, and ****p < 0.0001.

## Results

### HIF-2α expressed by lymphocytes might be negatively correlated with UC severity

**Figure 1:**
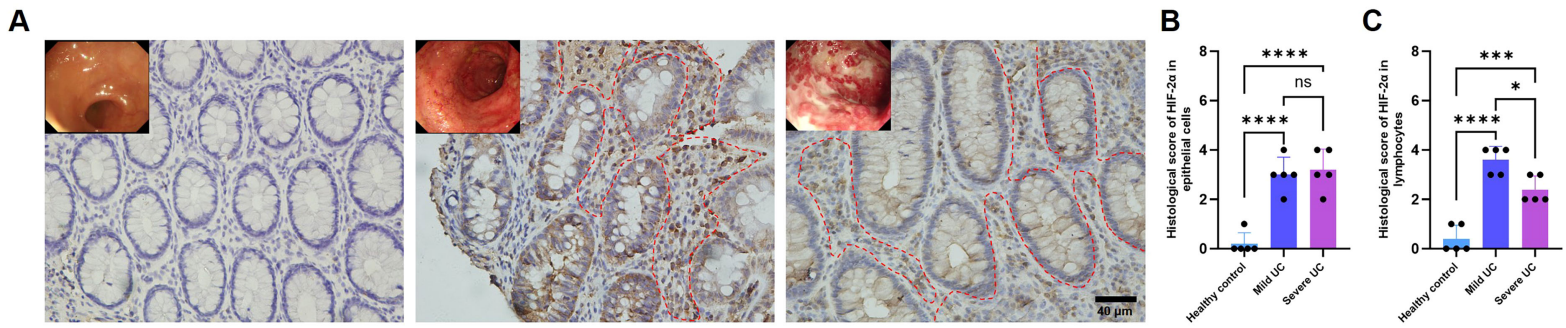
The expression of lymphocyte HIF-2α might be negatively correlated with UC severity.**(A)** IHC staining of HIF-2α in intestinal tissues of healthy controls, mild UC patients, and severe UC patients (scale bar, 100 μm). **(B)** Histological score of HIF-2α in submucosal epithelial cell (n = 5). **(C)** Histological score of HIF-2α in submucosal lymphocytes (n = 5). The region enclosed by the red dotted line indicates lymphocytes. The significance of the difference was ascertained using one-way analysis of variance (ANOVA) followed by Tukey’s *post-hoc* test. Asterisks denote statistical significance levels: * indicates p < 0.05, *** indicates p < 0.001, and **** indicates p < 0.0001.

Limited knowledge suggests that HIF-2α has complex and context dependent roles in intestinal pathology, showing not universally protective or isolated deleterious activities. To explore its function in UC, we evaluated intestine biopsies from 15 patients with active UC (divided to mild or severe) and normal volunteers. Immunohisto-chemical study and histological examination showed localization of HIF-2α in both intestinal epithelial cells and submucosal lymphocytes. Expression levels were significantly elevated in UC patient tissues relative to healthy controls (**Figures 1A-C**). Notably, however, submucosal lymphocytes from severe UC cases showed markedly reduced HIF-2α expression compared to those from mild UC subjects (**Figure 1C**). This inverse correlation between lymphocyte-specific HIF-2α levels and the extent of mucosal injury suggests that HIF-2α in lymphocytes may perform a regulatory role in limiting UC progression to severe disease.

### The T/NKT-HIF-2α cKO in T/NK T cells enhanced Th2 differentiation

**Figure 2:**
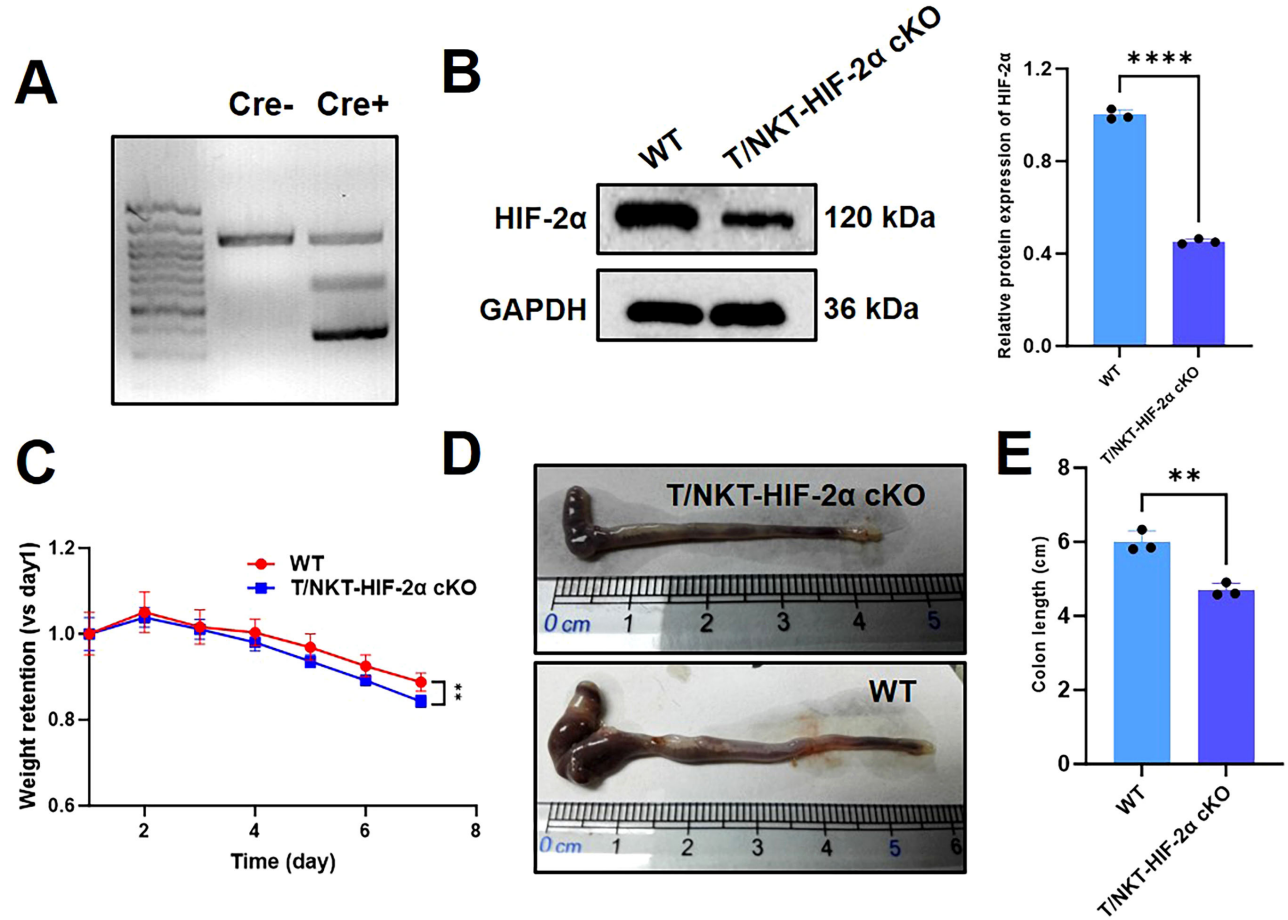
Impact of HIF-2α deficiency on DSS-induced intestinal inflammation in mice. **(A)** Polymerase Chain Reaction (PCR) outcomes regarding the disruption of the Hypoxia-Inducible Factor-2α (HIF-2α) gene in T and Natural Killer T (NKT) cells were obtained using Lck-Cre transgenic mice. **(B)** Western blotting analysis demonstrated a decrease in HIF-2α expression levels in DSS-treated T/NKT-HIF-2α cKO mice when compared to DSS-treated WT mice (n = 3). **(C)** The body weight retention was analyzed between T/NKT-HIF-2α cKO and WT mice after treatment with DSS for 7 days (n = 3). **(D)** Representative photographs of colons from T/NKT-HIF-2α cKO and WT mice were presented. **(E)** The colon lengths were measured and a significant decrease was observed in T/NKT-HIF-2α cKO mice (n = 3). The significance of the differences was assessed using ANOVA along with Tukey’s *post hoc* test and Student’s t test. **p < 0.01 and ****p < 0.0001 denote significant differences.

In order to begin an examination of the roles played by lymphocyte HIF-2α during inflammation, we generated a mouse model with T/NKT cell-specific deletion of HIF-2α via Cre-loxP-mediated recombination (4) (**Figure 2A**). Efficient knockout was validated by decreased expression levels of HIF-2α in colonic tissues of mutant mice (**Figure 2B**). After inducing colitis by DSS treatment, HIF-2α-deficient mice exhibited more pronounced body weight loss (**Figure 2C**), higher DAI index (**Supplementary Figure 3**) and greater colon shortening (**Figures 2D, E**) than WT controls, collectively indicating exacerbated intestinal pathology.

**Figure 3:**
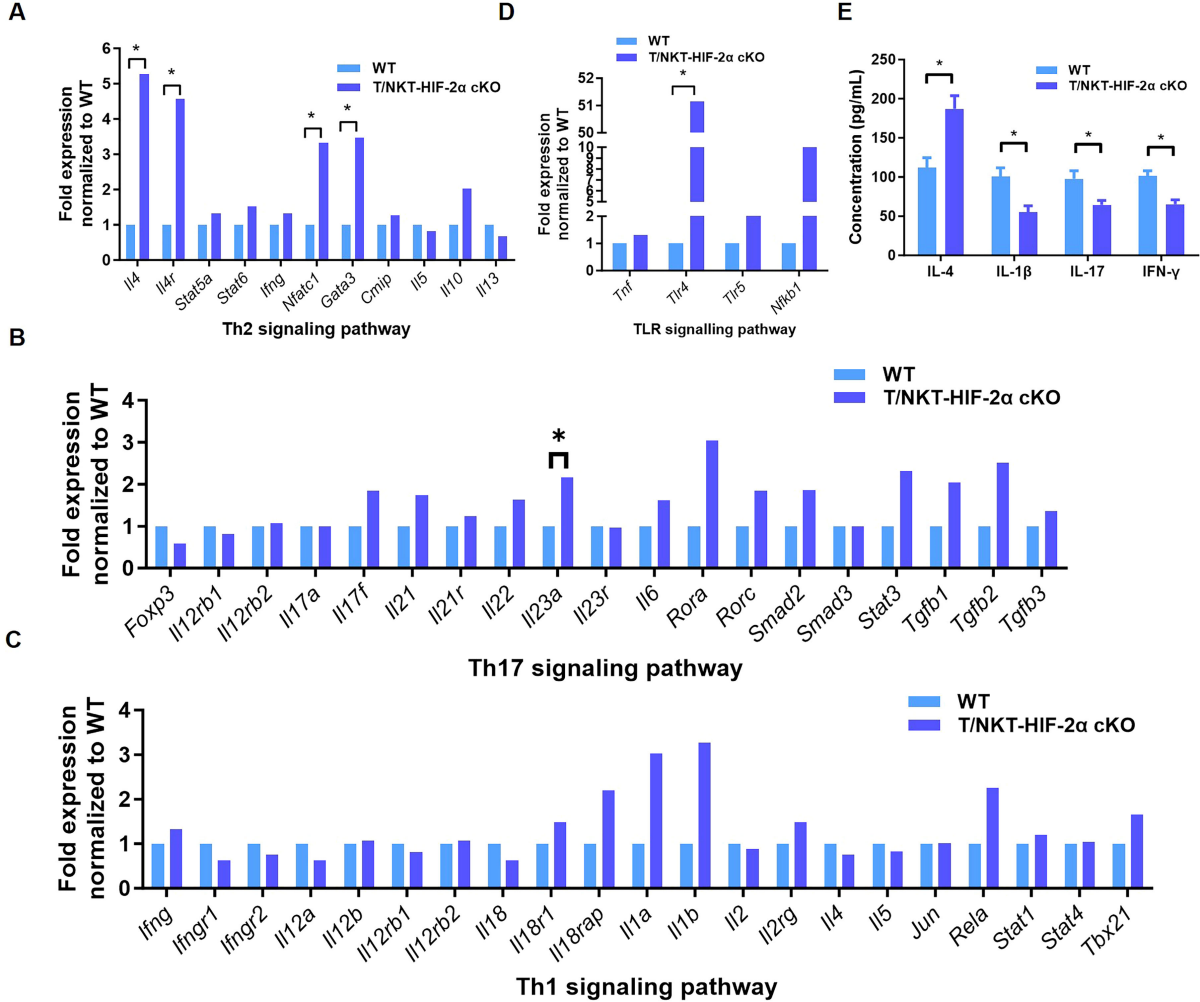
HIF-2α knockout promotes Th2 differentiation. The alteration in the expression of genes linked to the Th2 signaling pathway **(A)**, Th17 signaling pathway **(B)**, Th1 signaling pathway **(C)**, and TLR signaling pathway **(D)** was measured (n = 3) **(E)**. Enzyme-linked immunosorbent assay (ELISA) was used to measure the concentrations of IL-4, IL-1β, IL-17, and IFN-γ in the serum of T/NKT-HIF-2α cKO mice and WT mice. The Student’s t-test was utilized to assess the significance of the differences. A p-value of less than 0.05 (*p < 0.05) was deemed significant.

Due to the significance of T cell polarization in inflammatory bowel diseases development, and exacerbated phenotype upon HIF-2α ablation, we assumed that HIF-2α regulates T helper cell differentiation. We concentrated on profiling Th1, Th2, and Th17 transcriptional pathways by PCR array analysis. This resulted in substantial induction of major Th2-associated genes, Il4, Gata3, Il4ra, and Nfatc1 in the knockout mice versus WT littermates (**Figure 3A**). Significantly, Gata3 encodes the master Th2 transcription factor, and Il4 represents a canonical Th2 cytokine, suggesting that HIF-2α loss promotes Th2 lineage commitment.

In addition, the expression of Il23a associated with Th17 differentiation was also increased in HIF-2α-deficient mice (**Figure 3B**), indicating the concomitant induction of Th17-associated inflammation. While trended increases in the vast majority of Th1-related genes did not achieve significance, upward trends for Il1a, Il1b, Il18rap, and Rela (**Figure 3C**), together with marked induction of Tlr4 in Toll-like receptor signaling (**Figure 3D**), imply broader innate immune activation. Consistent with the transcriptional shift toward Th2 responses, serum IL-4 concentrations were significantly elevated in T/NKT-HIF-2α cKO mice, whereas IL-1β, IL-17, and IFN-γ levels were reduced (**Figure 3F**). Taken together, these data support a model wherein HIF-2α deficiency exacerbates experimental colitis, at least in part, by skewing T cell differentiation toward the Th2 lineage.

### HIF-2α deficiency promotes Th2 differentiation of CD4^+^ T cells *in vitro*

**Figure 4:**
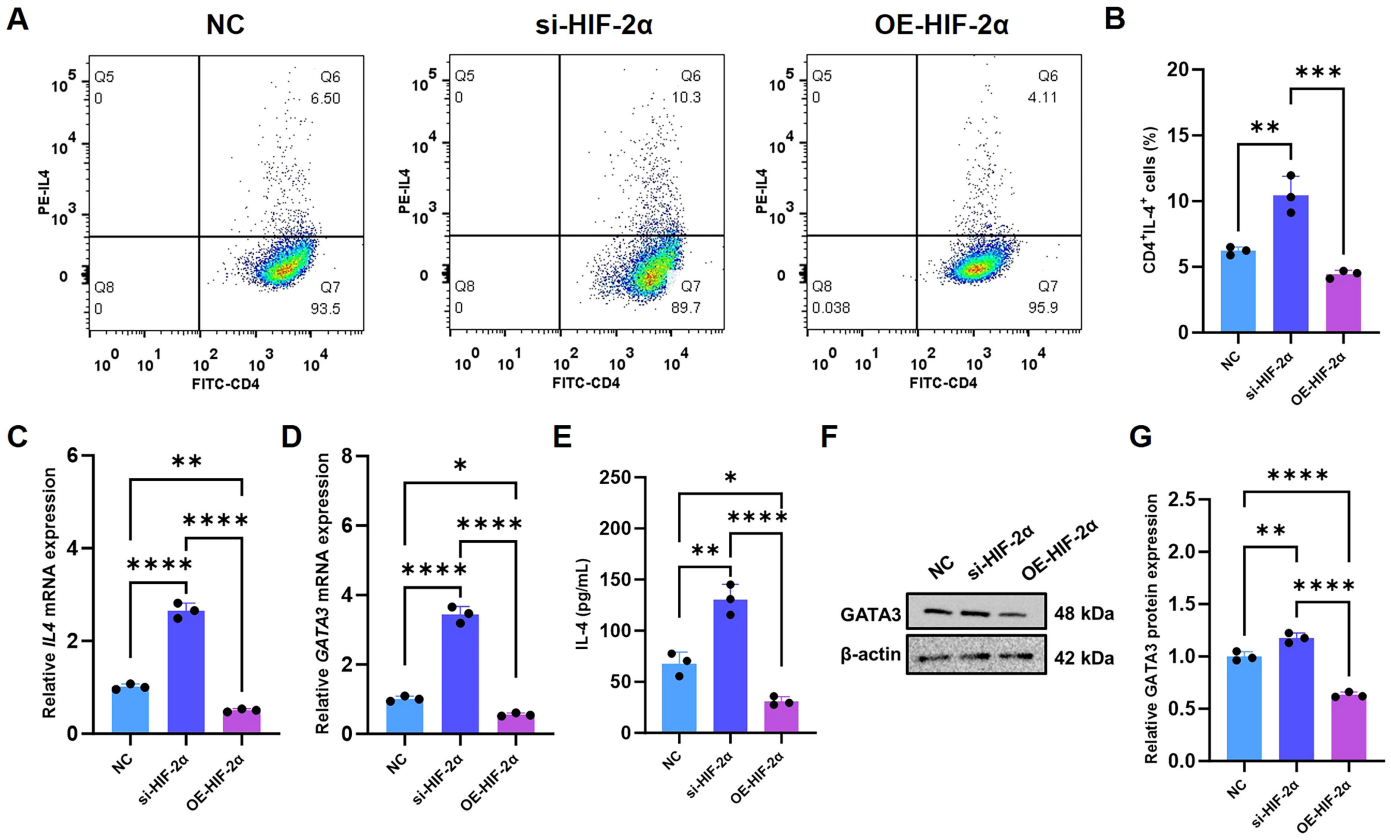
HIF-2α deficiency promotes Th2 cell differentiation. **(A)** The percentage of CD4^+^IL-4^+^ T cells was measured using flow cytometry (n = 3). **(B)** A statistical assessment was conducted on the CD4^+^IL-4^+^ T cells presented in **(A)**. The relative levels of mRNA expression for Il4 **(C)** and **(D)** Gata3 were determined (n = 3). **(E)** The quantity of IL-4 in the supernatant was detected via ELISA (n = 3). **(F)** The relative levels of protein expression for GATA3 were analyzed, and **(G)** a statistical evaluation of the gray values of the bands was carried out (n = 3). The significance of differences was ascertained through one-way ANOVA with Tukey’s *post-hoc* test. *p < 0.05, **p < 0.01, ***p < 0.001, ****p < 0.0001.

To establish a causal relationship between HIF-2α deficiency in lymphocytes, Th2 polarization, and exacerbated colitis, we focused on CD4^+^ T cells and investigated the underlying cell-intrinsic mechanisms by modulating HIF-2α expression via lentiviral transduction (**Supplementary Figure 4**). Consistent with our proposed model, HIF-2α knockdown significantly increased the frequency of IL-4-producing CD4^+^ T cells (**Figures 4A, B**), indicating enhanced Th2 differentiation. Furthermore, transcriptional and protein analyses confirmed that HIF-2α silencing robustly elevated expression of the canonical Th2 markers IL-4 and GATA3 (**Figures 4C–G**). In contrast, forced HIF-2α expression produced the opposite effect, effectively normalizing all observed Th2-promoting phenotypes.

### HIF-2α directly binds to Notch1-NICD and inhibits Notch-mediated Th2 differentiation

**Figure 5:**
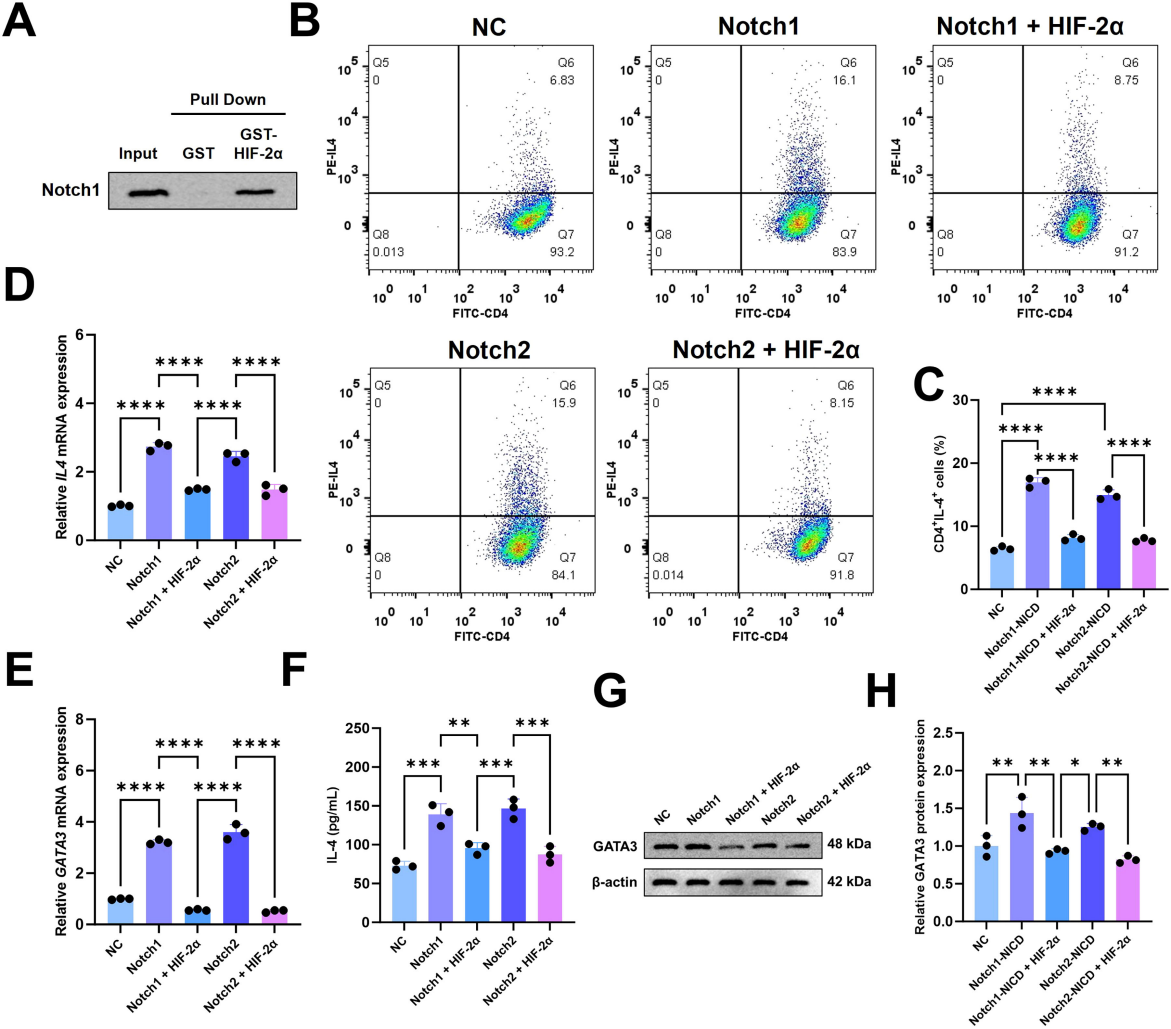
HIF-2α modulate NOTCH signaling pathway. **(A)** Pull-down assay showing the binding of HIF-2α and Notch1-NICD. **(B)** The proportion of Th2 (CD4^+^IL-4^+^) cells was detected by flow cytometry in different groups. These groups included the negative control (NC), Notch1 group, Notch2 group, Notch1+HIF-2α group, and Notch2+HIF-2α group. **(C)** Statistical analysis of the Th2 cell proportion presented in **(B)**; n = 3 mice per group. Subsequently, the mRNA levels of **(D)** Il4 and **(E)** Gata3 in various treatment groups were determined with n = 3 replicates per group. **(F)** The level of IL-4 in the supernatants from different groups was measured with n = 3. **(G, H)** Protein expression of GATA3 was analyzed in the groups. The differences within the groups were evaluated by one-way ANOVA and Tukey’s *post hoc* test. Significance levels were denoted as **p < 0.01, ***p < 0.001, and ****p < 0.0001.

The Notch signaling is an archetypical regulatory framework of cell fate such as proliferation, survival, differentiation and apoptosis, known for its essential functions in immune cell development including T lymphocyte differentiation. We first explored the possibility of crosstalk between HIF-2α and Notch signaling according to our observation that HIF-2α regulates Th2 differentiation in CD4^+^ T cells. By glutathione S-transferase (GST) pull-down assay with recombinant GST-HIF-2α fusion protein, we demonstrated that HIF-2α directly interacts physically with the active form of NICD, raising a potential means for crosstalk (**Figure 5A**).

We then transduced CD4^+^ T cells with lentiviral vectors encoding Notch1-NICD and Notch2-NICD, to create five experimental conditions: Negative Control (NC), Notch1-NICD, Notch2-NICD, Notch1-NICD + HIF-2α, and Notch2-NICD + HIF-2α. Both Notch paralogs robustly increased the IL-4-producing CD4^+^ T population compared to the NC group (**Figures 5B, C**), with no significant difference between the Notch1 and Notch2. However, co-overexpression of HIF-2α effectively reversed this NICD-driven Th2 polarization. Molecular analysis showed that HIF-2α co-expression resulted in abrogation of Notch-mediated transcriptional upregulation of Il4 and Gata3 (**Figures 5D, E**), with corresponding reductions in IL-4 secretion (**Figure 5F**) and GATA3 protein abundance (**Figures 5G, H**), collectively establishing HIF-2α as a potent inhibitor of Notch-induced Th2 commitment.

### HIF-2α inhibits Notch signaling pathway

**Figure 6:**
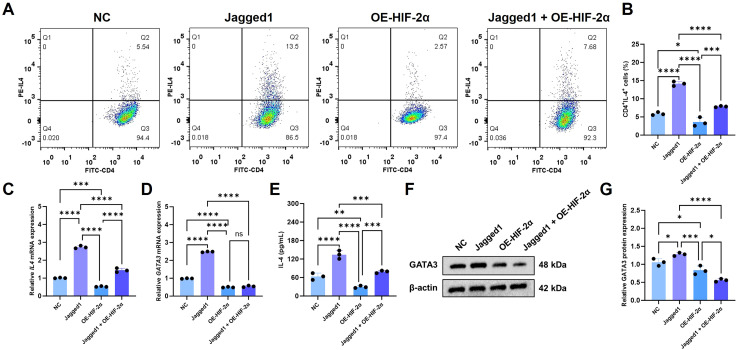
HIF-2α inhibits Th2 differentiation by suppressing Notch signaling pathway. The ratio of Th2 (CD4^+^IL-4^+^) cells **(A)** and the statistical assessment of Th2 cells across various treatments (n = 3) are presented. The mRNA expression levels of Il4 **(C)** and Gata3 **(D)** are also shown. Moreover, the concentration of IL-4 in the supernatants of different groups (n = 3) is depicted **(E)**. In addition, the protein expression levels of GATA3 in different groups (n = 3) are presented **(F, G)**. The significance of differences was ascertained via one-way ANOVA along with Tukey’s *post hoc* test. The symbols *p < 0.05, **p < 0.01, ***p < 0.001, and ****p < 0.0001 denote the levels of significance.

Jagged1, a prototypical Notch receptor ligand, is involved in activating the Notch pathway to promote commitment of Th2 cells. Flow cytometric analysis of Th2 differentiation by different treatment (**Figures 6A, B**) revealed that recombinant Jagged1 can largely and significantly expand the Th2 (CD4^+^IL-4^+^) population, indicating enhanced expression of Notch ligand was able to promote the polarization of Th2 by through Notch. However, this Jagged1-mediated effect was effectively suppressed by HIF-2α overexpression. To further characterize HIF-2α-mediated inhibition of Notch signaling, we quantified IL-4 and GATA3 expression at transcriptional (**Figures 6C, D**) and translational (**Figures 6E–G**) levels. Both mRNA and protein analyses confirmed that HIF-2α overexpression counteracts Jagged1-induced upregulation of these key Th2 markers, supporting a model where HIF-2α constrains Th2 differentiation through negative regulation of Notch signal transduction.

### HIF-2α deficiency further activates Notch signaling pathway and promotes Th2 differentiation, thereby exacerbating colitis *in vivo*

**Figure 7:**
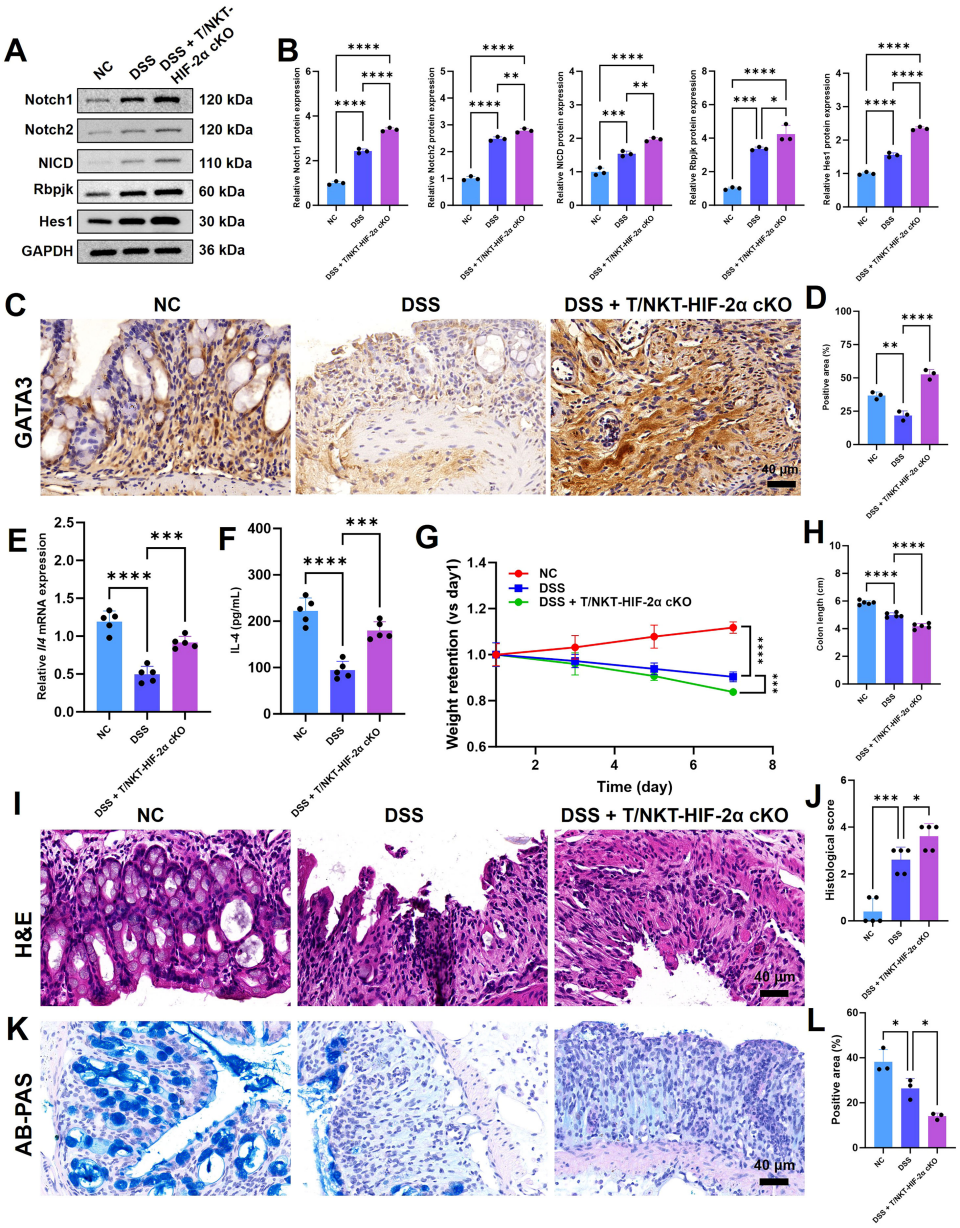
HIF-2α deficiency activates Notch signaling pathway and exacerbates colon injury. **(A)** Western blotting of Notch1, Notch2, NICD, Rbpjk and Hes1, and **(B)** the corresponding statistical analyses (n = 3). Glyceraldehyde-3-phosphate dehydrogenase (GAPDH) served as the loading control. **(C)** Immunohistochemical (IHC) staining was carried out to detect GATA3 in the colon tissues of the mice (scale bar: 40 μm). Subsequently, a statistical analysis was performed on the area where GATA3 was positive (n = 3). **(E)** The mRNA expression level and **(F)** the concentration of IL-4 in colon tissues of the mice (n = 5). **(G)** The weight retention and **(H)** the colon length of the mice in different groups (n = 5). **(I)** Typical hematoxylin and eosin (H&E)-stained pictures of colon tissues from various groups are presented (scale bar: 40 μm), and **(J)** shows the statistical assessment of histological scores (n = 3). **(K)** AB-PAS staining images of colon tissues from different groups (scale bar, 40 μm) and **(L)** the statistical analysis of the positive area (n = 3). The significance of the difference was ascertained via one-way ANOVA followed by Tukey’s *post-hoc* examination. *p < 0.05, **p < 0.01, ***p < 0.001, ****p < 0.0001.

Our *in vitro* results demonstrated that HIF-2α limits Th2 differentiation by inhibiting Notch signaling. We then wanted to confirm this regulatory axis *in vivo*. Immunoblot analysis of colonic tissues from DSS-treated mice revealed elevated protein levels of core Notch pathway components—including Notch1, Notch2, NICD, RBPJK, and Hes1—compared to normal controls, confirming pathway activation during colitis. This activation was further amplified in HIF-2α-deficient animals (**Figures 7A, B**). Consistent with the potentiation of Notch signaling, HIF-2α knockout increased expression of the Th2 markers GATA3 (**Figures 7C, D**) and IL-4 (**Figures 7E, F**) in colitic tissues.

Physiologically, HIF-2α deficiency accelerated the course of disease evidenced by enhanced weight loss (**Figure 7G**) and pronounced colon shortening (**Figure 7H**). H&E staining showed that the colon damage was worse in knockout mice (**Figures 7I, J**), and AB-PAS staining showed a more serious depletion of goblet cells (**Figures 7K, L**). Together, these *in vivo* findings support our model that HIF-2α loss of function increases Notch signaling to drive Th2-associated immunopathology and tissue injury during experimental colitis.

## Discussion

Here, we demonstrate a protective role for T cell-intrinsic HIF-2α in ulcerative colitis by repression of Notch-driven Th2 polarization. While total HIF-2α is increased in colonic tissue from UC patient colonic tissues, its level within lymphocyte populations shows an inverse relationship with clinical disease severity, indicating site-specific activity. Mice with conditional ablation of HIF-2α in T/NKT cells developed more severe DSS-induced colitis, accompanied by enhanced Th2 differentiation marked by elevated Il4 and Gata3 expression and increased IL-4 production.

Mechanistically, we identified a direct molecular interaction between HIF-2α and the activated NICD, through which HIF-2α constrains Notch signaling activation. Both cell-based and animal experiments confirmed that HIF-2α overexpression inhibits Jagged1-triggered Th2 differentiation, whereas genetic HIF-2α deficiency amplifies Notch pathway activity and Th2 responses, culminating in exacerbated colonic injury.

These findings offer a possible explanation for previous discrepancies concerning the role played by HIF-2α’s in intestinal inflammation. Although differentiating between pro-inflammatory and protective effects (18, 21, 22), our data establish its cell type-specific action in lymphocytes as a crucial immunomodulatory mechanism. Such an inverse correlation between lymphocyte HIF-2α levels and disease severity in human samples was also demonstrated, reinforcing its protective role within adaptive immunity.

The Notch pathway is established as a critical determinant of T cell fate (29, 30), with its dysregulation implicated in various inflammatory conditions (31). It is noteworthy that in our DSS model, although the Notch signaling pathway is activated during colitis (**Figures 7A, B**), this activation does not lead to a simultaneous substantial upregulation of GATA3 and IL-4 in wild-type mice (**Figures 7C–F**). This may seem contradictory to the classic Notch-Th2 theory (32–34). However, DSS-induced colitis is a model dominated by innate immunity and Th1/Th17-type immune responses (35, 36), rather than Th2-type. In this intensely pro-inflammatory environment, even when Notch signaling is activated, its classic function of promoting Th2 differentiation may be overridden or suppressed by more powerful microenvironmental signals that inhibit Th2 (37). High levels of cytokines, such as IFN-γ, IL-6, in the microenvironment strongly drive Th1 and Th17 differentiation (38, 39), and directly or indirectly inhibit the expression and function of GATA3, thereby suppressing Th2 differentiation. Furthermore, Th2 responses may become significant only in later stages of colitis or under specific conditions. In this study, HIF-2α expressed in lymphocytes acts as a ‘molecular brake’ on Notch signaling, limiting the conversion of Notch signals into a Th2 differentiation program by directly binding to and inhibiting the activity of NICD. Therefore, in wild-type animals with intact HIF-2α function, even if Notch signaling is upregulated, its pro-Th2 effects are restrained. Only in the absence of HIF-2α can Notch signaling fully unleash its ability to drive Gata3 and IL-4 expression and promote Th2 polarization, leading to more severe colitis pathology. This mechanism emphasizes that in complex pathological environments like inflammatory bowel disease, the final output of key signaling pathways (such as Notch) is highly dependent on the context-specificity provided by intrinsic cellular regulators (such as HIF-2α). Although Th2 differentiation represented the most prominently affected pathway in T/NKT-HIF-2α cKO mice, we also noted moderate alterations in Th17 and innate immune signaling, indicating broader immunomodulatory capacity. The predominant Th2 phenotype, however, underscores its centrality in HIF-2α-mediated protection against colitis.

This study originated from observations of HIF-2α expression profiles in lymphocytes from the colonic tissues of ulcerative colitis patients and employed gene knockout mice targeting T/NKT lymphocytes for functional validation. Although the animal model encompassed NKT cells, given that CD4^+^ T cells are the precursor cells for Th2 differentiation, our subsequent mechanistic investigations were primarily focused on this specific lymphocyte subset. Consequently, the precise impact of HIF-2α on various NKT cell subsets under disease conditions was not explored in the current work, representing a direction for future research. Moreover, our study confirms the existence of a direct physical interaction between HIF-2α and NICD. This binding is crucial for HIF-2α-mediated suppression of the Notch signaling pathway and the subsequent restriction of Th2 cell differentiation. However, the precise molecular mechanism by which HIF-2α functionally inhibits NICD activity still requires further elucidation. Our preliminary data suggest that HIF-2α may interfere with the formation of the NICD transcriptional complex. This indicates that HIF-2α likely suppresses the transcriptional activity of NICD primarily after its nuclear entry, potentially by interfering with its interaction with co-activators or DNA-binding elements, rather than by promoting its degradation or preventing its nuclear import. This hypothesis needs to be further validated and refined through more in-depth structural biology studies and functional experiments. Clarifying this precise inhibitory mechanism will contribute to a more comprehensive understanding of the context-specific role of HIF-2α in lymphocytes.

Although the Lck-Cre system enables T/NKT cell-specific gene deletion, it is important to acknowledge its potential limitations. Lck-Cre expression begins during early thymocyte development, which may lead to gene deletion in immature T cells and potentially affect thymic selection or peripheral T cell homeostasis. In our study, however, the observed exacerbation of DSS-induced colitis in HIF-2α cKO mice was accompanied by enhanced Th2 polarization and Notch signaling in colitic tissues, suggesting that the phenotype is likely driven by the loss of HIF-2α in mature peripheral T cells rather than developmental alterations. While we cannot entirely rule out off-target effects in non-T cells due to low-level Cre expression in other lineages, our validation by Western blot and flow cytometry confirmed efficient and specific HIF-2α knockdown in CD4^+^ T cells. Future studies using inducible Cre systems could further dissect the temporal role of HIF-2α in established colitis.

Additionally, several methodological considerations warrant acknowledgment. (1) Although DSS-induced colitis model is commonly used, it does not exemplify chronic relapsing-remitting characteristic of human UC, which may pose constriction on direct applicability to translation. (2) Despite that Lck-Cre-mediated deletion targets T/NKT cells, potential off-target effects or influences during early T cell development cannot be entirely excluded. (3 *In vitro* experiments under normoxia might not completely recapitulate the hypoxic microenvironment of inflamed intestine, and thus it is needed to investigate HIF-2α-Notch crosstalk under physiological oxygen tension. (4) The small size of human cohort (n = 15) limits applicability of correlation between HIF-2α expression and severity of disease. (5) Although we demonstrated that HIF-2α directly bound to the NICD, details regarding how binding mainly affects NICD stability, nuclear translocation, or transcriptional complex assembly need more structural and functional characterization.

Notwithstanding these limitations, our findings provide compelling evidence supporting T cell HIF-2α as a key regulator of Th2 differentiation and a promising therapeutic target for ulcerative colitis.

## Conclusion

Collectively, our work defines HIF-2α as a pivotal suppressor of Th2 cell differentiation in T lymphocytes, functioning through direct constraint of Notch signal transduction. Genetic ablation of HIF-2α results in aggravated intestinal inflammation driven by unrestrained Th2 polarization. These results not only resolve prior contradictions regarding HIF-2α’s function in ulcerative colitis but also nominate it as a promising molecular target for therapeutic intervention in pathogenic T cell responses associated with inflammatory bowel disease. Subsequent investigations should focus on evaluating the clinical relevance of modulating the HIF-2α-Notch regulatory axis for treating UC and related immune pathologies.

## Data Availability

The original contributions presented in the study are included in the article/**Supplementary Material**, further inquiries can be directed to the corresponding author/s.
