# Supplementary material for: T cell-specific HIF-2α attenuates colitis by antagonizing notch-driven Th2 differentiations
Source: Front Immunol. 2026 Mar 3;17:1755068. doi: 10.3389/fimmu.2026.1755068 (PMC12992320; doi:10.3389/fimmu.2026.1755068)
Supplement: Supplementary file 1 [file Table1.docx]

**T Cell-Specific HIF-2α Attenuates Colitis by Antagonizing Notch-Driven Th2 Differentiations**

**Ting Gao^1#^, Liangfeng Gao^1#^, Hui Zhang^2#^, Zaizhi Liu^1^, Qing Zhu^1^, Chunyan Wang^1*^, Song Zhang^1*^, Nan Feng^1*^**


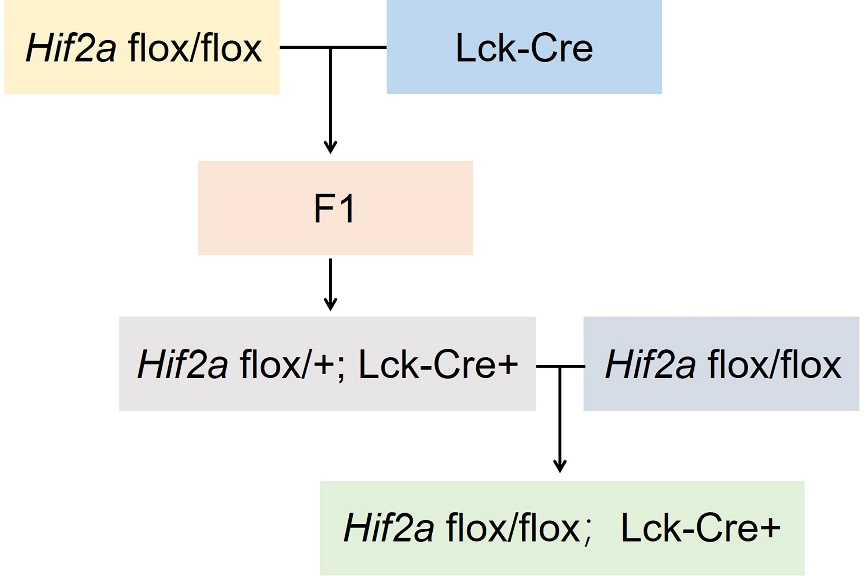


**Supplementary Figure S1.** The generation steps of T/NKT-HIF-2α cKO mice. To generate T/NKT-HIF-2α cKO mice, the following experimental steps are performed. First, parental mice are prepared: *Hif2a* flox/flox mice (homozygous for the floxed allele) and *Lck-Cre* transgenic mice (expressing Cre recombinase under a T-cell-specific promoter). These mice are crossbred to produce the F1 generation. From the F1 offspring, individuals with the genotype *Hif2a* flox/+; *Lck-Cre*+ are selected. These F1 carriers are then backcrossed with *Hif2a* flox/flox mice. From the resulting offspring, mice with the target genotype *Hif2a* flox/flox; *Lck-Cre*+ are screened and used as the experimental group (T/NKT cell-specific HIF-2α knockout). Littermates that are *Hif2a* flox/flox but do not carry the Cre transgene (*Lck-Cre* negative) are used as wild-type controls. Finally, genotyping is confirmed via PCR analysis of genomic DNA extracted from tail or ear clips.

**^
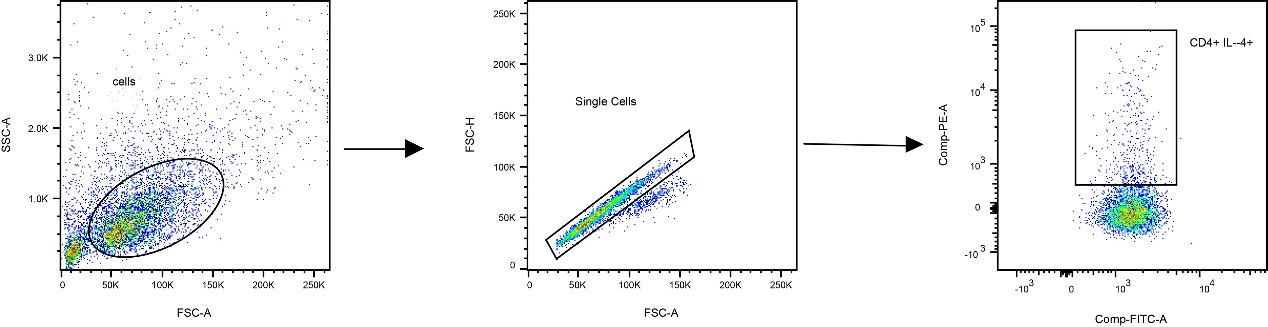
^**

**Supplementary Figure S2:** The flow cytometry gating strategy diagram of CD4⁺IL-4⁺ cells.


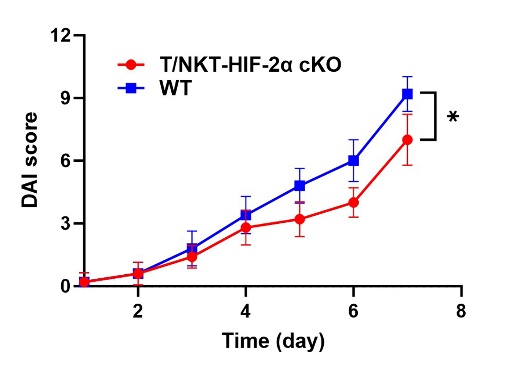


**Supplementary Figure S3.** The DAI score of the DSS-induced WT and T/NKT-HIF-2α cKO mice (n = 3).

**Supplementary Figure S4.** The overexpression and silencing efficiency of HIF2α (n = 3). The significance of the difference was ascertained via one-way ANOVA followed by Tukey's post-hoc examination. *p < 0.05, ***p < 0.001, ****p < 0.0001.
